# Characterization of association of human mitochondrial lysyl-tRNA synthetase with HIV-1 Pol and tRNA_3_^Lys^

**DOI:** 10.1186/s12858-018-0092-x

**Published:** 2018-03-21

**Authors:** Fawzi Khoder-Agha, José M. Dias, Martine Comisso, Marc Mirande

**Affiliations:** Institute for Integrative Biology of the Cell (I2BC), CEA, CNRS, Univ. Paris-Sud, Université Paris-Saclay, 1 avenue de la Terrasse, 91190 Gif-sur-Yvette, France

**Keywords:** HIV-1, Integrase, Transframe (TF or p6*), Mitochondrial lysyl-tRNA synthetase, Binding affinity, tRNA_3_^Lys^ packaging

## Abstract

**Background:**

An important step in human immunodeficiency virus type 1 (HIV-1) replication is the packaging of tRNA_3_^Lys^ from the host cell, which plays the role of primer RNA in the process of initiation of reverse transcription. The viral GagPol polyprotein precursor, and the human mitochondrial lysyl-tRNA synthetase (mLysRS) from the host cell, have been proposed to be involved in the packaging process. More specifically, the catalytic domain of mLysRS is supposed to interact with the transframe (TF or p6*) and integrase (IN) domains of the Pol region of the GagPol polyprotein.

**Results:**

In this work, we report a quantitative characterization of the protein:protein interactions between mLysRS and its viral partners, the Pol polyprotein, and the isolated integrase and transframe domains of Pol. A dissociation constant of 1.3 ± 0.2 nM was determined for the Pol:mLysRS interaction, which exemplifies the robustness of this association. The protease and reverse transcriptase domains of GagPol are dispensable in this association, but the TF and IN domains have to be connected by a linker polypeptide to recapitulate a high affinity partner for mLysRS. The binding of the viral proteins to mLysRS does not dramatically enhance the binding affinity of mLysRS for tRNA_3_^Lys^.

**Conclusions:**

These data support the conclusion that the complex formed between GagPol, mLysRS and tRNA_3_^Lys^, which involves direct interactions between the IN and TF domains of Pol with mLysRS, is more robust than suggested by the previous models supposed to be involved in the packaging of tRNA_3_^Lys^ into HIV-1 particles.

## Background

Reverse transcription of human immunodeficiency virus type-1 (HIV-1) genomic RNA requires annealing of tRNA_3_^Lys^ to the primer binding site (PBS) [1, 2]. Indeed, the 18, 3’-OH terminal nucleotides of this host tRNA are complementary to the PBS sequence of viral RNA. The primer RNA is packaged into the virus during the assembly of the newly forming particles [3, 4]. Viral infectivity decreases with decreasing concentrations of tRNA_3_^Lys^ in the particles [5]. HIV-1 viruses also contain lysyl-tRNA synthetase (LysRS) from the host cell [6, 7]. Polyclonal antibodies directed to the cytoplasmic species of LysRS (cLysRS) identified a reactive polypeptide in extracts of HIV-1 virions [6]. But since a single gene encodes the cytoplasmic and mitochondrial species of LysRS by means of alternative splicing [8], the two LysRS species share 576 amino acid residues in common, among the 597 residues of cLysRS, and could not be distinguished by the antibodies used in that study. Monospecific antibodies directed to the very N-terminal peptides characteristic of cytoplasmic or mitochondrial LysRS revealed the presence of only the mitochondrial LysRS (mLysRS) species in extracts of HIV-1 virions purified from HIV-1 LAI strain cultured on CEM cells [7].

Human LysRS is a homodimer, with each monomer consisting of three distinct domains. Two domains are well conserved among species and are totally identical in human cLysRS and mLysRS. These are the C-terminal part of the enzyme, made of about 360 residues, which corresponds to its dimerization and catalytic domain, and the anticodon binding domain, made of about 170 residues, which is appended to the N-terminus of the catalytic domain. In human, the crystal structure of this core enzyme has been solved [9]. Human cLysRS possesses an additional, eukaryote-specific N-terminal polypeptide extension, made of about 70 residues, which is a functional tRNA-binding domain that stabilizes the tRNA:LysRS complex [10, 11]. In the mature form of mLysRS, after cleavage of the very N-terminal, 30 residue mitochondrial targeting sequence, this domain is in part conserved and also binds tRNA [12]. In human, cLysRS associates with 8 other aminoacyl-tRNA synthetases and with the 3 auxilliary proteins p18, p38 and p43 to form the multi-synthetase complex (MSC), which is a strictly cytoplasmic entity [13]. Its association is mediated by the scaffold protein p38, also named AIMP2 [14, 15].

The HIV-1 Gag and GagPol polyproteins, before they are proteolytically processed within mature particles, are essential players in virion assembly [16]. The Gag polyprotein consists in the matrix (MA), capsid (CA), nucleocapsid (NC) and p6 domains; the GagPol polyprotein, produced after a translational − 1 frameshifting event occurring between NC and p6, also carries the transframe (TF or p6*), protease (Pr), reverse transcriptase (RT) and integrase (IN) domains. Association of mLysRS with HIV-1 GagPol polyprotein was observed in vitro and is believed to be responsible for its packaging into viral particles. It involves the catalytic domain of the synthetase and the transframe and integrase domains of the Pol region of GagPol [17]. No other viral protein seems to be required to form the tRNA_3_^Lys^ packaging complex, consisting of GagPol, mLysRS and tRNA_3_^Lys^ [18]. Earlier studies based on the packaging of cLysRS suggested that monomeric cLysRS, after dissociation of the dimer, associates with the CA domain of Gag via its dimerization interface [19, 20].

In this work, we characterized in vitro the association of mLysRS with the Pol domain of GagPol, and more specifically with the TF and IN domains of the Pol region of GagPol. We also analyzed the binding of tRNA_3_^Lys^ to mLysRS in the absence or presence of the viral partners of mLysRS. The quantitative data obtained in this study support the conclusion that the HIV-1 tRNA_3_^Lys^ packaging complex is much more stable than previously anticipated, and would require proteolytic processing of GagPol to release the primer RNA into the virions.

## Methods

### Expression of HIV-1 pol in insect cells

The Pol coding region from pNL4-3 (nucleotides 2091 to 5093) was introduced between the *Eco*RI and *Xho*I sites of pFastBac1 (Life Technologies). A His_6_ tag coding sequence has been appended at the C-terminus of Pol. Recombinant bacmids and baculoviruses were obtained as previously described [18]. Baculoviruses were used to infect 2 l of High Five cells (Life Technologies) grown in suspension in Express Five SFM medium (Life Technologies). After 56 h of culture at 27 °C, cells were harvested by centrifugation, washed with ice-cold buffer 150/10 (20 mM K-phosphate pH 7.5, 150 mM NaCl, 10 mM imidazole, 5% glycerol, 5 mM 2-mercaptoethanol), and the cell pellet was stored at − 80 °C. Cells were lysed at 37 °C after addition of 30 ml of buffer 150/10 containing 1% Triton X-100, in the presence of 1 mM Pefabloc, 10 mM benzamidine and 10 mM PMSF. After addition of 60 ml of buffer 500/50 (20 mM K-phosphate pH 7.5, 500 mM NaCl, 50 mM imidazole, 5% glycerol, 5 mM 2-mercaptoethanol), extract was clarified by centrifugation at 70,000 *g* for 30 min at 4 °C and incubated 1 h at 4 °C with 0.5 ml of Ni-NTA Superflow matrix (Qiagen). Beads were extensively washed with buffer 500/50, and elution was performed by adding 5 × 1 ml of buffer 500/400 (20 mM K-phosphate pH 7.5, 500 mM NaCl, 400 mM imidazole, 5% glycerol, 5 mM 2-mercaptoethanol). Eluate was concentrated by ultrafiltration (Vivaspin 6, 10 kDa) to a volume of 0.5 ml and applied to an TSK G4000 SW column (300 × 7.5 mm) equilibrated in 20 mM Hepes pH 6.8, 250 mM NaCl, 2% glycerol and 2 mM DTT. Fractions containing Pol were concentrated by ultrafiltration (Vivaspin 6, 10 kDa), and stored at − 80 °C. Protein concentration was determined by using a calculated absorption coefficient of 1.786 *A*_280_ units mg^− 1^ cm^2^.

### Expression of HIV-1 integrase in insect cells

The IN coding region from pNL4-3 (nucleotides 4230 to 5093) was introduced between the *Eco*RI and *Xho*I sites of pFastBac1 (Life Technologies). A His_6_ tag coding sequence has been appended at the C-terminus of integrase. Recombinant bacmids and baculoviruses were obtained as previously described [18]. Baculoviruses were used to infect 8 l of High Five cells (Life Technologies) grown in suspension in Express Five SFM medium (Life Technologies). After 68 h of culture at 27 °C, cells were harvested by centrifugation, washed with ice-cold buffer 150/10, and the cell pellet was stored at − 80 °C. Cells were lysed at 37 °C after addition of 120 ml of buffer 150/10 containing 1% Triton X-100, in the presence of 1 mM Pefabloc, 10 mM benzamidine and 10 mM PMSF. After addition of 240 ml of buffer 500/50, extract was clarified by centrifugation at 70,000 *g* for 30 min at 4 °C and incubated 1 h at 4 °C with 1 ml of Ni-NTA Superflow matrix (Qiagen). Beads were extensively washed with buffer 500/50, and elution was performed by adding 5 × 1 ml of buffer 500/400. Eluted proteins were dialyzed against buffer ASU (20 mM Tris-HCl pH 7.0, 150 mM NaCl, 1 M urea, 10% glycerol, 1 mM EDTA, 10 mM 2-mercaptoethanol) and applied to a Mono S HR 5/5 column (GE Healthcare) equilibrated in the same buffer. Proteins were eluted by a linear gradient (40 column vol.) of NaCl from 150 to 450 mM. Fractions containing integrase were concentrated by ultrafiltration (Vivaspin 6, 10 kDa), dialyzed against storage buffer (20 mM K-phosphate pH 7.5, 1 M NaCl, 2 mM DTT), and stored at − 80 °C. Protein concentration was determined by using a calculated absorption coefficient of 1.529 *A*_280_ units mg^− 1^ cm^2^.

### Expression of HIV-1 transframe in *E. coli*

The transframe (TF) coding region from pNL4-3 (nucleotides 2091 to 2252) was codon-optimized for expression in *E. coli* and introduced between the *Nco*I and *Xho*I sites of pET28b. A His_6_ tag coding sequence has been appended at the C-terminus of TF. Expression of TF was conducted in *E. coli* BL21(DE3) (Invitrogen) grown at 37 °C in 8 l of LB medium supplemented with kanamycin (50 μg/ml). When the culture reached an *A*_600_ = 0.3, temperature was adjusted to 20 °C and expression was induced by addition of 1 mM IPTG for 4 h when *A*_600_ was equal to 0.5. Cells were washed twice with ice-cold buffer 150/10, resuspended in 60 ml of the same buffer containing 1 mM Pefabloc, 10 mM benzamidine and 10 mM PMSF, and lysed by sonication. All subsequent steps were conducted at 4 °C. Extract was clarified by centrifugation at 70,000 g for 30 min and incubated 1 h at 4 °C with 1 ml of Ni-NTA Superflow matrix (Qiagen). Beads were extensively washed with buffer 500/50, and elution was performed by adding 5 × 1 ml of buffer 500/400. Eluted proteins were dialyzed against buffer AS (20 mM Tris-HCl pH 7.5, 10 mM KCl, 10% glycerol, 10 mM 2-mercaptoethanol), and applied to a Mono S HR 5/5 column equilibrated in the same buffer. Proteins were eluted by a linear gradient (40 column vol.) of KCl from 10 to 300 mM. Fractions containing TF were concentrated by ultrafiltration (Amicon Ultra-4, 3 kDa), dialyzed against PBS (136 mM NaCl, 2.7 mM KCl, 8 mM Na_2_HPO_4_, 1.47 mM KH_2_PO_4_), and stored at − 80 °C. Protein concentration was determined by using a calculated absorption coefficient of 0.773 *A*_280_ units mg^− 1^ cm^2^.

The apparent native molecular mass of TF was determined by gel filtration on a Yarra 3u SEC-2000 column (300 × 4.6 mm) (Phenomenex) equilibrated in 200 mM K-phosphate (pH 6.8), 5 mM 2-mercaeptoethanol, and developed at room temperature at a flow rate of 0.05 ml/min. The calibration curve was established by using cytochrome C, ovalbumin, and bovine serum albumin as marker proteins. For a particular protein, its elution was described in term of the corresponding *K*_av_ value*. K*_av_ *= (V*_e_*-V*_0_*)/(V*_t_*-V*_0_*),* where *V*_e_ is the elution volume of the particular molecule, *V*_0_ the void volume of the column, and *V*_t_ the total bed volume. *V*_0_ and *V*_t_ were determined with dextran blue (> 5 MDa) and vitamin B12 (1.35 kDa), respectively.

### Expression of TF-Sx-IN surrogates of pol in *E. coli*.

The TF and IN sequences from pNL4-3 were introduced into pET28b with a *Bam*HI site between the TF and IN coding regions. The *Bam*HI site encodes for a 2 residue spacer, S2 (Gly-Ser), leading to the expression of the TF-S2-IN-H6 fusion protein. An oligonucleotide duplex (5’-GATCTGGGGGTGGCG and 5’-GATCCGCCACCCCCA, encoding a GGGGS peptide) was recursively introduced into the *Bam*HI site to give pET28b/TF-S7-IN-H6 (one insert), pET28b/TF-S12-IN-H6 (two inserts), pET28b/TF-S17-IN-H6 (three inserts) and pET28b/TF-S22-IN-H6 (four inserts).

Expression of the TF-Sx-IN fusion proteins was conducted in *E. coli* BL21(DE3) (Invitrogen) grown at 37 °C in 6 l of LB medium supplemented with kanamycin (50 μg/ml). When the culture reached an *A*_600_ = 0.5, expression was induced by addition of 1 mM IPTG for 4 h. Cells were washed twice with ice-cold buffer 150/10, resuspended in the same buffer (1 ml per g of cell pellet) containing 1 mM Pefabloc, 10 mM benzamidine and 10 mM PMSF, and lysed in an Eaton Press after freezing in dry ice. All subsequent steps were conducted at 4 °C. After addition of 2 vol. of buffer 150/10, extracts were clarified by sonication and by centrifugation at 70,000×*g* for 30 min. After incubation 1 h at 4 °C with 1 ml of Ni-NTA Superflow matrix (Qiagen), beads were extensively washed with buffer 500/50, and elution was performed by adding 5 × 1 ml of buffer 500/400. Eluted proteins were dialyzed against buffer 20 mM Tris-HCl pH 7.5, 100 mM NaCl, 1 M urea, 10% glycerol, 1 mM EDTA, 10 mM 2-mercaptoethanol, and applied to a Mono S HR 5/5 column (GE Healthcare) equilibrated in the same buffer. Proteins were eluted by a linear gradient (40 column vol.) of NaCl from 100 to 400 mM. Fractions containing the TF-Sx-IN fusion proteins were adjusted to 0.02% Triton X-100, concentrated by ultrafiltration (Vivaspin 6, 10 kDa), dialyzed against storage buffer (20 mM Tris-HCl pH 7.5, 250 mM NaCl, 5% glycerol, 10 mM 2-mercaptoethanol, 0.02% Triton X-100), and stored at − 80 °C. Protein concentration was determined by using the BioRad Protein Assay.

### Antibodies and western blot analysis

Rabbit anti-TF antibodies were generated against a synthetic peptide (KAREFSSEQTRANSPTRRE) corresponding to residues 10-28 of HIV-1 transframe protein (Life Technologies). Western blot analyses were conducted with goat anti-rabbit secondary antibodies conjugated with peroxidase (Chemicon) and the SuperSignal West Pico chemiluminescent substrates (Thermo Scientific).

### HTRF assay

Homogeneous time-resolved fluorescence (HTRF) assays were performed in black, half-area, 96-well microplates. Mitochondrial LysRS (mLysRS) or a derivative with a C-terminal deletion of 22 aminoacid residues (mLysRS∆C) were expressed in *E. coli* with a C-terminal HA-tag (YPYDVPDYA), and purified as described [12]. mLysRS-HA (1.5 nM, dimer concentration) was incubated with various concentrations of Pol-H^6^ (0.02 to 10 nM, dimer concentration), IN-H^6^ (0.5 to 125 nM, dimer concentration) or TF-H6 (1 to 1000 nM, monomer concentration) in 10 mM Tris-HCl pH 7.5, 50 mM NaCl, 10 mM 2-mercaptoethanol and BSA at 1 mg/ml, for 1 h on ice. For the determination of the binding affinities of the TF-Sx-IN surrogates, the buffer was supplemented with 0.02% Triton X-100. Antibodies (Cisbio) directed to the His-tag, conjugated with Eu^3+^ cryptate, and to the HA-tag, conjugated with XL665, were added and incubation was continued for 30 min. After addition of 50 mM KF, fluorescence of Eu^3+^ cryptate and of XL665 was recorded at 620 nm (*I*_620_) and 665 nm (*I*_665_), respectively, after excitation of Eu^3+^ cryptate at 317 nm, in an Infinite M1000 PRO microplate reader (TECAN). Results are expressed as the ratio of *I*_665_/*I*_620_.

### Circular dichroism spectroscopy

CD spectra were recorded at 20 °C or 90 °C with a path length of 1 mm in a Jasco J-810 apparatus equipped with a Peltier temperature controller. Each spectrum is a mean of 10 scans. Protein was dialyzed against 25 mM K-phosphate (pH 7.5) and its final concentration was 12 μM. Spectra were analyzed using the Dichroweb software [21].

### Fluorescence polarization assay

Fluorescence polarization was measured in an Infinite M1000 Pro reader (TECAN) after incubation for 1 h on ice in PBS buffer containing 0.02% Triton X-100. Mitochondrial LysRS (mLysRS) was expressed in insect cells and purified as described [12]. tRNA_3_^Lys^-Cy3 was synthesized in vitro with Cyanine3 attached at the 5′-extremity (eurofins). Renaturation of tRNA was performed in 5 mM MgCl_2_ after heating at 90 °C for 2 min, and slow cooling at 25 °C. The efficiency of renaturation was measured in the tRNA aminoacylation reaction (420 pmol/*A*_260_).

## Results

### Characterization of the interaction between Pol and mLysRS

**Fig. 1:**
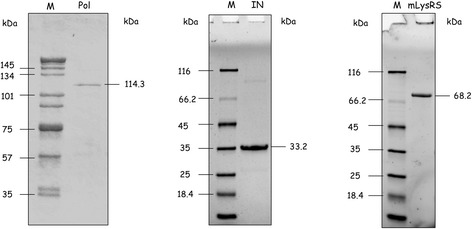
Analysis of the purified proteins by SDS-PAGE. The Pol sub-domain of the GagPol polyprotein (Pol), the integrase domain of Pol (IN), and the mitochondrial species of human LysRS (mLysRS), expressed in insect cells and purified as described in “Materials and Methods”, were analyzed by SDS-polyacrylamide gel electrophoresis. Size markers (M) were run in parallel. Molecular masses are indicated in kDa

**Fig. 2:**
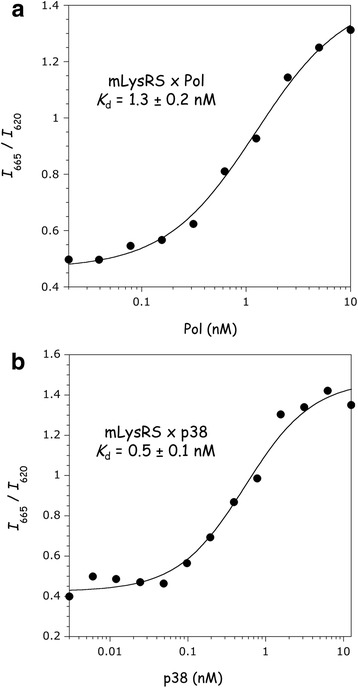
Association of mLysRS with Pol and p38. The binding affinities of mLysRS to Pol (**a**) or p38 (**b**) were determined in an HTRF assay using 1.5 nM (**a**) or 0.5 nM (**b**) of HA-tagged mLysRS and increasing concentrations of His-tagged Pol or p38, all expressed as dimer concentrations. Experimental values (symbols) were fit (curves) to a binding equation assuming that one dimer of Pol or p38 binds one dimer of mLysRS. The binding constants and the associated standard deviations result from three independent measurements

We previously established that the catalytic domain of the mitochondrial species of human LysRS interacts with the Pol domain of the polyprotein GagPol from HIV-1 [17]. Human mLysRS was expressed in *E. coli* with a C-terminal HA-tag, and the Pol polyprotein was expressed in insect cells with a C-terminal His-tag (Fig. 1). A homogeneous time-resolved fluorescence (HTRF) assay [22] was designed to measure the robustness of the interaction between the two proteins. In this type of FRET assay, an antibody is labeled with the energy donor (Europium, Eu^3+^-cryptate), and a second antibody is labeled with the acceptor (XL665, a phycobiliprotein pigment). An anti-H^6^-Eu^3+^ antibody and an anti-HA-XL665 antibody were used to characterize the mLysRS/Pol complex. After excitation at 317 nm, fluorescence was recovered at 620 nm (Eu^3+^) and at 665 nm (XL665). The HTRF signal corresponds to the ratio of the intensity recovered at 665 nm to the intensity at 620 nm (*I*_665_/*I*_620_). The binding curve obtained for the mLysRS/Pol complex is shown in Fig. 2a. An apparent dissociation constant *K*_d_ of 1.3 ± 0.2 nM was determined.

To validate this assay, we also measured the affinity between mLysRS and the scaffold protein of the MSC, p38. Indeed, we previously showed that mLysRS and cLysRS, the mitochondrial and cytoplasmic species of LysRS, are equally able to interact in vitro with p38. This interaction involves the catalytic domain of LysRS which is identical in mLysRS and cLysRS [17]. In this case, the HTRF assay was performed using an anti-HA-Eu^3+^ antibody and an anti-H^6^-XL665 antibody. As shown Fig. 2b, the binding affinity determined by this HTRF assay for the complex formed between mLysRS and p38 (*K*_d_ of 0.5 ± 0.1 nM) is very similar to that previously determined by surface plasmon resonance (SPR; *K*_d_ of 0.3 ± 0.2 nM) [23].

### Characterization of the interaction between the TF and IN domains of Pol and mLysRS

**Fig. 3:**
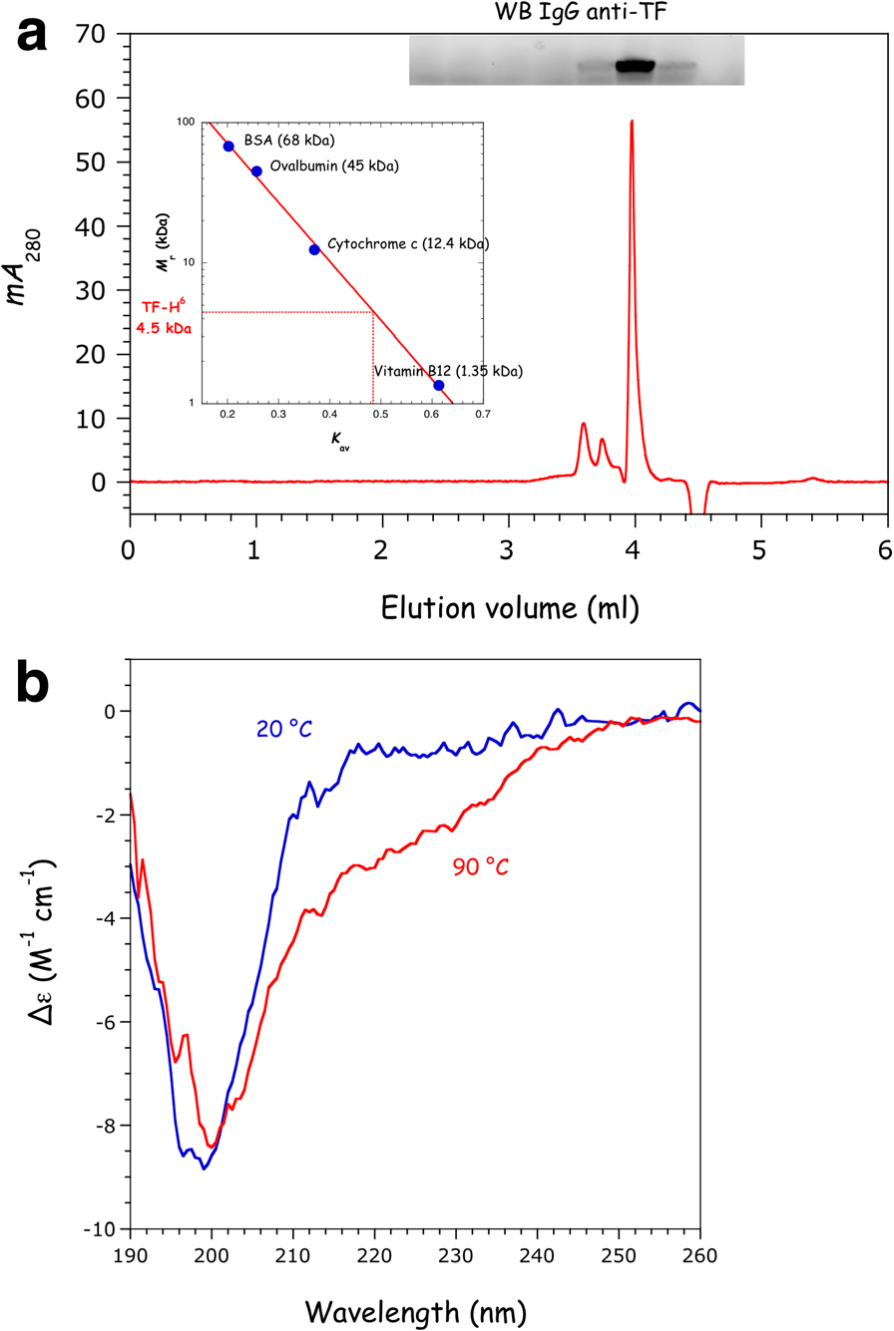
Characterization of TF expressed in *E. coli*. The transframe (TF, also known as p6*) domain of Pol was expressed in *E. coli* with a C-terminal His-tag. **a** Purified TF was subjected to size exclusion chromatography on a Yarra SEC-2000 column as described under “Materials and Methods”. Elution of TF was followed at 280 nm, and confirmed by Western blotting with anti-TF antibodies. The apparent molecular mass of TF was deduced from its relative elution volume *K*_av_ (inset). **b** CD spectra of TF at 20 °C and 90 °C

The transframe (TF, also named p6*) and integrase (IN) domains of Pol are the two domains of the polyprotein that interact with the catalytic domain of mLysRS. IN was expressed in insect cells with a C-terminal His-tag (Fig. 1). TF was expressed in *E. coli* with a C-terminal His-tag. This small, 54-amino acid residue protein does not share sequence similarity with any other protein registered in the data libraries, and its three-dimensional structure is not known. The purified protein, unambiguously detected with specific antibodies, was eluted as a symmetrical peak on a size-exclusion column (Fig. 3a) with an apparent *M*_r_ of 4.5 kDa, compatible with a compact, globular, monomeric protein in solution. Analysis of the purified protein by circular dichroism revealed a low content in secondary structure elements, with an estimate of 2% and 6% of residues folded into α-helices or β-strands, respectively (Fig. 3b). Accordingly, heat denaturation at 90 °C was only followed by a minor change of its CD spectrum (Fig. 3b).

**Fig. 4:**
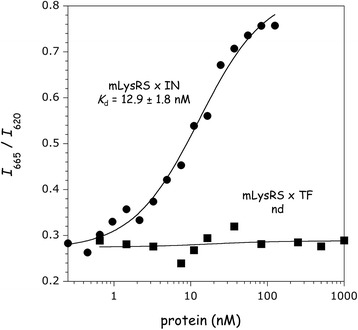
Association of mLysRS with IN and TF. The binding affinities of mLysRS to IN or TF were determined in the HTRF assay using 1.5 nM of HA-tagged mLysRS and increasing concentrations of His-tagged interacting proteins, as described in Fig. 2. The binding constant of mLysRS to TF could not be determined (nd)

Using the HTRF assay described above with the anti-H^6^-Eu^3+^ and anti-HA-XL665 antibody pair, an about 10-fold higher *K*_d_ of 12.9 ± 1.8 nM was determined for the mLysRS/IN complex (Fig. 4), as compared to the mLysRS/Pol complex. The binding affinity for the mLysRS/TF complex could not be determined using this assay (Fig. 4).

### Designing a surrogate of Pol

**Fig. 5:**
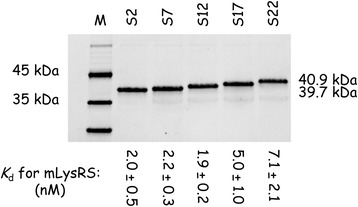
Association of the TF-Sx-IN surrogates of Pol with mLysRS. The fusion proteins with spacers made of 2 (S2), 7 (S7), 12 (S12), 17 (S17) or 22 (S22) amino acids were analyzed by SDS-PAGE and their binding affinities for mLysRS were determined in the HTRF assay, as described in Fig. 2. The binding constants and the associated standard deviations result from three independent measurements

The TF and IN domains of Pol were identified by the 2-hybrid system as the domains interacting with the catalytic domain of mLysRS, and no interaction with the protease (Pr) and reverse transcriptase (RT) domains of Pol were detected [17]. The Pol polyprotein cannot be obtained in large amount, and at high concentration. To overcome this problem, we designed a surrogate lacking the Pr and RT domains. The two mLysRS-binding domains of Pol were fused by a spacer domain. The catalytic domain of mLysRS is about 60 Å in length. Five fusion proteins with spacers of different sizes were constructed. A spacer made of 2 (5 Å), 7 (23 Å), 12 (41 Å), 17 (59 Å) or 22 (77 Å) amino acid residues was inserted between the TF and IN domains, to give the TF-Sx-IN proteins, Sx being S2, S7, S12, S17 or S22. It is made of Gly and Ser residues to promote good flexibility and more easily accommodate the presumed binding sites of TF and IN on mLysRS. These fusion proteins were expressed in *E. coli* with a C-terminal His-tag, and purified to homogeneity (Fig. 5).

Apparent dissociation constants *K*_d_ of these Pol derivatives for mLysRS were measured using the HTRF assay in the conditions of Fig. 4. The TF-S2-IN, TF-S7-IN and TF-S12-IN constructs were found to be the best surrogates of Pol, with *K*_d_ of 2.0 ± 0.5, 2.2 ± 0.3 and 1.9 ± 0.2 nM, respectively. These values were less than 2-fold higher than that determined for Pol (1.3 nM, Fig. 2a). The two other constructs, TF-S17-IN and TF-S22-IN also did bind mLysRS, but with a more than 2-fold higher *K*_d_ of 5.0 ± 1.0 and 7.1 ± 2.1 nM. Because linking the TF and IN domains with a spacer as short as 5 Å (*K*_d_ 2.0 nM) is able to restore an affinity close to that observed for the native Pol polyprotein (*K*_d_ 1.3 nM), it can be inferred that the binding sites of IN and TF on mLysRS are located in close proximity, less than 5 Å apart. When the spacer is too long, its flexibility is not sufficient to accommodate the two proteins on mLysRS.

### Association of tRNA_3_^Lys^ within the packaging complex

**Fig. 6:**
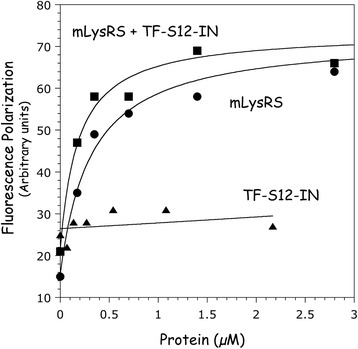
Analysis of tRNA-binding by fluorescence polarization. The binding affinity of mLysRS for tRNA_3_^Lys^ was measured using 50 nM of Cy-3 labeled tRNA_3_^Lys^, in the absence or in the presence of 0.5 μM of TF-S12-IN. TF-S12-IN alone did not bind tRNA_3_^Lys^. The deduced binding constants and the associated standard deviations result from at least three independent measurements

The binding affinity of mLysRS for tRNA_3_^Lys^ carrying a Cy-3 fluorescent probe at its 5′-extremity was measured at equilibrium using a fluorescence polarization assay. An apparent dissociation constant *K*_d_ of 315 ± 89 nM was deduced from the binding curve (Fig. 6). This binding constant is consistent with the *K*_d_ of 250 ± 40 nM determined by a gel retardation assay [12].

The binding affinity of tRNA_3_^Lys^ for mLysRS was also determined in the presence of the TF-S12-IN construct (Fig. 6). TF-S12-IN alone did not bind tRNA_3_^Lys^ (Fig. 6). The binding constant measured for association of tRNA_3_^Lys^ with the mLysRS/TF-S12-IN complex, *K*_d_ of 185 ± 60 nM, was 2-fold lower than that determined for mLysRS alone. Thus, association of TF-S12-IN to mLysRS slightly stabilizes association of tRNA_3_^Lys^ with mLysRS.

**Fig. 7:**
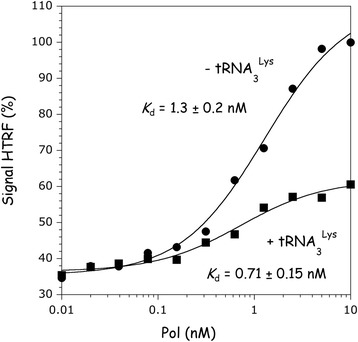
Association of Pol with mLysRS in the presence of tRNA_3_^Lys^. The binding affinity of Pol to mLysRS was determined in the absence or in the presence of 1 μM tRNA_3_^Lys^, in the HTRF assay as described in Fig. 2. The binding constants and the associated standard deviations result from three independent measurements

In parallel, the binding affinity of Pol for mLysRS was monitored in the absence or in the presence of tRNA_3_^Lys^ (Fig. 7). A binding constant of 0.71 ± 0.15 nM was determined for association of Pol with mLysRS in the presence of tRNA_3_^Lys^, as compared to 1.3 ± 0.2 nM in the absence of tRNA. In the presence of tRNA_3_^Lys^, the amplitude of the HTRF signal was 3-fold lower. This means that the energy transfer between the two fluorophores is less efficient, which suggests that the distance between them is increased upon binding of tRNA_3_^Lys^ to mLysRS. Therefore, we tested the possibility that the C-terminal peptide of mLysRS, which is likely to be mobile and on which one of the two fluorophores is attached, could be involved in tRNA and/or integrase binding.

### Comparison of mLysRS and mLysRS∆C for their association with tRNA or IN

**Fig. 8:**
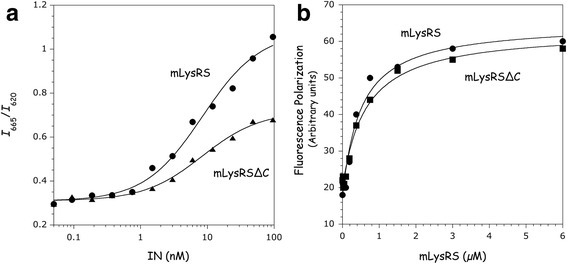
Comparison of mLysRS and mLysRS∆C for binding to IN and to tRNA_3_^Lys^. mLysRS and mLysRS∆C were expressed in *E. coli* with a C-terminal HA-tag. The HTRF assay, conducted as described in the legend of Fig. 4, was used to compare their binding constants to IN (**a**), and fluorescence polarization, conducted as described in the legend of Fig. 6, was used to compare their affinities for Cy-3 labeled tRNA_3_^Lys^ (**b**). The binding constants and the associated standard deviations result from three independent measurements

Human LysRS possesses a C-terminal, 22-amino acid residue polypeptide extension which is absent in the bacterial enzyme. The HA-tag is fused at the C-terminus of this eukaryotic-specific appended domain, which is supposed to be flexible and was not observed in the crystal-structure of human LysRS [9]. Using the HTRF assay described in Fig. 4 to monitor association of mLysRS with IN, similar dissociation constants were measured for the mLysRS/IN (*K*_d_ of 11.8 ± 1.9 nM) and mLysRS∆C/IN (*K*_d_ of 8.3 ± 1.1 nM) complexes (Fig. 8a). The amplitude of the HTRF signal was also decreased by a factor 1.9 in the assay with mLysRS∆C, consistent with the different location of the HA-tag in the native and truncated enzymes. The binding affinity of mLysRS and mLysRS∆C, containing the C-terminal HA-tag, for tRNA_3_^Lys^ was determined using the fluorescence polarization assay (Fig. 8b). mLysRS∆C binds tRNA with a *K*_d_ of 630 ± 121 nM, as compared to 489 ± 123 nM for the full-length enzyme. Thus, the very C-terminal peptide of human mLysRS does not seem to be involved in either tRNA or integrase binding.

## Discussion

We previously showed that mLysRS interacts with the Pol region of the GagPol polyprotein, and that aspartyl-tRNA synthetase, another Class II synthetase closely evolutionary related to LysRS and displaying a high level of sequence similarity, did not [17]. In this work, we characterized the interaction of mLysRS with Pol, and observed that this association is robust, with an apparent dissociation constant of 1.3 nM (Fig. 2), which is a strong suggestion of specificity. By comparison, interaction of LysRS with the scaffold protein p38 of the stable cytoplasmic multi-synthetase complex [23] is achieved with a binding constant of 0.5 nM (Fig. 2). The value of 1.3 nM should be compared with the equilibrium dissociation constant of 310 nM previously reported for association of cLysRS with Gag [19]. Further characterization of cLysRS/Gag interaction suggested that helix 4 from the CTD (C-terminal domain) of HIV-1 CA, located outside the dimeric interface of CA but involved in hexameric and pentameric assemblies of CA [24], and helix H7 of human cLysRS, located in the Motif 1 dimerization domain of LysRS, were involved in this interaction [20, 25]. It is noteworthy that human tryptophanyl-tRNA synthetase (TrpRS), a Class I synthetase completely unrelated to LysRS, displaying a structural organization and a dimer interface essentially distinct, also interacted with Gag with a binding constant of 1550 nM, only 5-fold higher than that determined for the cLysRS/CA complex. Because the protein interfaces of dimeric LysRS, dimeric TrpRS and helix 4 of CA mainly consist of several hydrophobic residues, the observed heterodimeric interactions could be the result of serendipitous associations, involving poorly complementary hydrophobic interfaces. We previously showed that mLysRS/Gag association could not be observed in conditions where mLysRS/Pol interaction was detected [17]. The high-affinity interactions measured in our study suggest that the complex made between mLysRS and Pol, which displays a 100-fold stronger affinity as compared with the cLysRS/Gag complex, corresponds to the specific and physiologically relevant interaction.

Our in vitro data are in agreement with observations made in vivo. It has long been known that GagPol is essential for the selectivity of tRNA^Lys^ incorporation into HIV-1 particles [26]. Deletion of reverse transcriptase and integrase sequences in Pol severely impairs the selectivity of this process. In Gag viral-like particles that do not contain GagPol, tRNA packaging is severely reduced [4, 26]. Several mutants of integrase with normal in vitro integrase activity, but yielding non infectious particles and reduced levels of viral DNA synthesis were described [27, 28], but the exact role of integrase in this process was not understood. HIV-1 integrase is essential for integration of viral DNA into host chromosome, but also fulfills other crucial roles related to virion morphogenesis [29]. In light of our data, it is also tempting to speculate that some of the previously described mutations could impair association of IN with mLysRS.

The HIV-1 capsid assembly has a fullerene-like organization made of hexameric and pentameric rings of CA [24]. According to the proposed capsid models, the Pol region of the GagPol polyprotein precursor, which accounts for about 5% of total Gag, is protruding from the internal side of immature virions, that will form the mature capsid core [30]. Thus, the Pol domain of GagPol would be readily accessible for binding the mLysRS:tRNA^Lys^ complex. By contrast, the atomic model of HIV-1 capsid assembly reveals that helix H4 from the CTD domain of CA is burried inside the structure [31], which precludes its interaction with LysRS. Such an interaction would inhibit the assembly of the particle.

The finding that association of GagPol with mLysRS and tRNA_3_^Lys^ is much more stable than previously thought also has a strong implication on the mechanism of release of tRNA into the viral particle. Maturation of the viral proteins, and especially the cleavage of GagPol into its individual proteins mediated by the viral protease Pr, is likely to be a prerequisite for the release of tRNA_3_^Lys^ to serve as a primer during the initiation step of reverse transcription.

The main contribution to the stability of the mLysRS/Pol complex is the interaction between mLysRS and IN, which displays a binding constant of about 12.9 nM (Fig. 4). TF is a compact and monomeric protein, but does not contain a significant amount of secondary structure elements, in agreement with NMR spectroscopy data [32]. Even if TF alone does not bind mLysRS with a binding constant strong enough to be measured using our HTRF assay, when IN and TF are carried on a single polypeptide, either linked by the Pr and RT sequences as in native GagPol protein, or linked by an artificial spacer of the appropriate size, the presence of TF strengthens association of the viral protein with mLysRS, the synergy of association leading to binding constants of 1.3 or 2.0 nM (Figs. 2 and 5), respectively. The finding that replacement of Pr and RT sequences by an artificial spacer does not significantly affect the binding of mLysRS and of tRNA_3_^Lys^ to the complex (Figs. 6 and 7) strongly argues that Pr and RT are dispensable in this process.

It is noteworthy that upon binding of tRNA_3_^Lys^ to mLysRS (Fig. 7), or after removal of the C-terminal polypeptide extension of mLysRS (Fig. 8a), the amplitude of the HTRF signal recovered upon binding of the viral partner decreases. Because the HTRF signal rests in part on the interaction of one of the two reporter antibody to the HA-tag appended at the C-terminus of mLysRS, this suggests that this peptide is subjected to important structural rearrangements upon tRNA binding. However, no difference in IN or in tRNA binding was observed between mLysRS and mLysRS∆C (Fig. 8). The structural organization of this peptide in human LysRS is not known, but could be similar to that observed for the equivalent peptide in *Entamoeba histolytica* LysRS [33]. In the crystal structure reported for this enzyme crystallized in the absence of tRNA, this peptide adopts an α-helix conformation. However, the place occupied by this peptide in the crystal is not compatible with the binding of a tRNA molecule [33]. This suggests that this peptide is mobile and may adopt different conformations relative to the core enzyme, in the absence or in the presence of a tRNA molecule. The function of this eukaryote-specific C-terminal extension in LysRS is not known, and its putative role in the functioning of LysRS in its translational or non-translational activity remains to be deciphered.

## Conclusions

It has been well established that the GagPol polyprotein precursor of HIV-1 is required for packaging of tRNA_3_^Lys^ into new viral particles; however, the cellular partner as well as the domains of GagPol involved in this process were controversial. In this study, we demonstrate that association of the mitochondrial species of human lysyl-tRNA synthetase with the integrase domain of GagPol is 100-fold more stable than the interactions previously reported in the assembly of the tRNA_3_^Lys^ packaging complex, which strongly argues for its relevance in this process.

## Abbreviations

AIMP2: Aminoacyl tRNA Synthetase Complex Interacting Multifunctional Protein 2
cLysRS: cytoplasmic LysRS
FRET: Fluorescence resonance energy transfer
HTRF: Homogeneous time-resolved fluorescence
LysRS: Lysyl-tRNA synthetase
mLysRS: mitochondrial LysRS
MSC: Multisynthetase complex
PBS: Primer binding site
TrpRS: Tryptophanyl-tRNA synthetase
